# Ciprofloxacin-Loaded Inhalable Formulations against Lower Respiratory Tract Infections: Challenges, Recent Advances, and Future Perspectives

**DOI:** 10.3390/pharmaceutics16050648

**Published:** 2024-05-11

**Authors:** Vijay Kumar Panthi, Kathryn E. Fairfull-Smith, Nazrul Islam

**Affiliations:** 1 Pharmacy Discipline, School of Clinical Sciences, Queensland University of Technology (QUT), Brisbane, QLD 4001, Australia; vijaykumar.panthi@hdr.qut.edu.au; 2 School of Chemistry and Physics, Faculty of Science, Queensland University of Technology (QUT), Brisbane, QLD 4001, Australia; k.fairfull-smith@qut.edu.au; 3 Centre for Materials Science, Queensland University of Technology (QUT), Brisbane, QLD 4001, Australia; 4 Centre for Immunology and Infection Control (CIIC), Queensland University of Technology (QUT), Brisbane, QLD 4001, Australia

**Keywords:** ciprofloxacin, formulations, lower respiratory tract infections, preclinical, clinical study

## Abstract

Inhaled ciprofloxacin (CFX) has been investigated as a treatment for lower respiratory tract infections (LRTIs) associated with cystic fibrosis (CF), chronic obstructive pulmonary disease (COPD), and bronchiectasis. The challenges in CFX effectiveness for LRTI treatment include poor aqueous solubility and therapy resistance. CFX dry powder for inhalation (DPI) formulations were well-tolerated, showing a remarkable decline in overall bacterial burden compared to a placebo in bronchiectasis patients. Recent research using an inhalable powder combining *Pseudomonas* phage PEV20 with CFX exhibited a substantial reduction in bacterial density in mouse lungs infected with clinical *P. aeruginosa* strains and reduced inflammation. Currently, studies suggest that elevated biosynthesis of fatty acids could serve as a potential biomarker for detecting CFX resistance in LRTIs. Furthermore, inhaled CFX has successfully addressed various challenges associated with traditional CFX, including the incapacity to eliminate the pathogen, the recurrence of colonization, and the development of resistance. However, further exploration is needed to address three key unresolved issues: identifying the right patient group, determining the optimal treatment duration, and accurately assessing the risk of antibiotic resistance, with additional multicenter randomized controlled trials suggested to tackle these challenges. Importantly, future investigations will focus on the effectiveness of CFX DPI in bronchiectasis and COPD, aiming to differentiate prognoses between these two conditions. This review underscores the importance of CFX inhalable formulations against LRTIs in preclinical and clinical sectors, their challenges, recent advancements, and future perspectives.

## 1. Introduction

Lower respiratory tract infections (LRTIs) pose life-threatening risks and can significantly affect health and the economy. Conditions such as cystic fibrosis (CF), chronic obstructive pulmonary disease (COPD), and bronchiectasis are commonly linked to LRTIs [1,2]. The primary agents behind these infections are diverse and include contagious bacteria of both Gram-positive and Gram-negative types, such as *Pseudomonas aeruginosa*, *Staphylococcus aureus*, *Streptococcus pneumoniae*, and *Burkholderia* species [3,4]. Various types of antibiotics are extensively used to manage LRTIs [5]. Depending on the severity of the infections, antibiotics are administered orally or parenterally. However, these conventional methods of antibiotic delivery necessitate significantly high systemic doses, potentially leading to inadequate drug levels in the lungs to effectively circumvent the infection and unwanted adverse effects [6,7].

Recently, inhaled antibiotics have been applied to manage LRTIs [6]. The rationale for administering antibiotics by inhalation is that it enables direct delivery of antibiotics to the airways at the infection site (targeted drug delivery), which can lead to elevated local drug concentrations, a swift onset of action, reduced adverse effects, and enhanced drug bioavailability [8]. For instance, when amikacin was administered through inhalation, the highest drug concentrations in bronchial secretions surpassed those in the bloodstream by a factor exceeding 1000 [9]. Conversely, IV delivery resulted in drug levels three times higher in the bloodstream compared to bronchial secretions. Consequently, directing amikacin to the lungs via inhalation created a >3000-fold difference in the concentration ratio between the lungs and the bloodstream relative to IV administration [9,10].

Ciprofloxacin (CFX) is a fluoroquinolone antibiotic with a demonstrated ability to attain elevated drug proportions in the lungs and lesser systemic drug levels when administered through inhalation than oral or IV routes [11]. Notably, the efficacy of IV CFX in treating LRTIs or severe pneumonia was frequently inadequate in patients colonized with *P. aeruginosa*. Instances included the inability to eliminate the organism, the persistence of infection, and the emergence of resistance [12]. Furthering this, the findings from the studies suggest that IV doses of 300 mg (or possibly up to 400 mg) every 12 h might lack efficacy in combating *P. aeruginosa* lung infections owing to insufficient drug levels in the lungs [11]. In cases of severe pneumonia where patients were colonized with *P. aeruginosa*, even elevated doses of IV CFX (400 mg every 8 h) were unsuccessful in achieving elimination and resulted in the development of resistance. Therefore, an inhaled CFX product, capable of attaining higher drug proportions in the lungs, holds the potential for increased effectiveness and a reduced risk of resistance development [11,12]. The CFX dry powder for inhalation (DPI) has two ionizable groups: a carboxylic acid and a secondary amine with pKa values of 6.09 and 8.74, respectively. In a low pH solution (around pH 4), both groups are protonated, giving CFX a net positive charge. However, in the higher pH environment of the lungs (around pH 7.4), the carboxylic acid group loses its proton, and CFX exists as a zwitterion, or betaine form, with a net neutral charge. The selection of the neutral (zwitterionic) form of CFX was crucial in developing CFX DPI. This form can exist in two dry forms (modification I and modification II) or four different hydrated forms (0.5 hydrate, 3.5 hydrate, 4.8 hydrate, and 6.0 hydrate), with changes between these states depending on temperature and relative humidity. The 3.5 hydrate form is primarily found in the drug product, leading to the drug substance being designated as CFX 3.5 hydrate or CFX betaine hydrate [13]. The hydrated (betaine) form of CFX has lower solubility (70 µg/mL at neutral pH) compared to the HCl salt, which is absorbed quickly through lung tissue. The lower solubility of the hydrated form enables a prolonged duration in the lungs [14]. CFX HCl is commonly utilized in oral and parenteral preparations. However, when inhaled orally, it has poor lung targeting and is quickly absorbed into the systemic circulation, with a half-life in rat lungs of less than 1 h, rendering it suboptimal for lung administration. An effective approach applied to enhance the lung residence duration of CFX HCl is to incorporate it in liposomes. Liposomal fabrications of CFX HCl demonstrate a longer duration in the lungs than non-encapsulated fabrications [13,15]. Additionally, studies have shown that the solubility of CFX complexed with mono-6-deoxy-6-aminoethylamino-β-cyclodextrin increased by 7.0-fold than pure CFX [16]. In addition, phase I studies investigating the pharmacodynamics (PD) and pharmacokinetics (PK) of CFX DPI have been conducted in healthy individuals, as well as in patients with CF and COPD. These investigations revealed that CFX DPI achieved higher lung concentrations while maintaining lower systemic exposure [17,18]. The drug concentrations in elevated sputum were notably higher than those in plasma. For example, in a study involving CF patients, a single regimen of CFX DPI (32.5 mg) led to maximum concentrations (C_max_) of 34.9 mg/L and 0.0790 mg/L, and an area under the curve (AUC) of 89.5 mg·h/L and 0.425 mg·h/L in sputum and plasma, respectively [19]. In vivo preclinical investigations also indicated that CFX DPI had a longer half-life compared to soluble CFX HCl, suggesting prolonged lung retention [14]. CFX HCl has a serum half-life of around 4 h, whereas in a phase I study with healthy volunteers, the terminal half-life of CFX DPI was determined to be 9.5 h [20].

**Figure 1:**
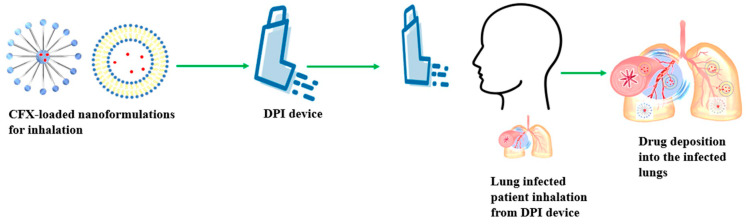
Schematic representation of CFX-loaded DPI formulations for pulmonary delivery.

For approximately three decades, CFX has been employed in managing several illnesses, including chronic otorrhea, LRT, endocarditis, skin and soft tissue, gastrointestinal, and urinary tract infections [21]. Currently, scientists are actively employing inhalable formulations incorporating CFX to assess its potential in efficiently addressing LRTIs (Figure 1). CFX, a second-generation quinolone, functions by inhibiting type 2 bacterial DNA topoisomerases, specifically DNA gyrase and topoisomerase IV, which are common sites of quinolone resistance. Currently, quinolones are prescribed for several infectious disorders, even though antimicrobial resistance (AMR) is becoming an issue against their efficacy. Moreover, no approved antibiotic treatments currently exist to decrease pulmonary exacerbations or halt disease advancement in patients suffering from LRTIs. For the past few years, CFX-loaded DPI formulations have demonstrated significant effectiveness against LRTIs in various clinical settings. However, three key unresolved issues necessitate further exploration: identifying the appropriate patient group, determining the best treatment duration, and accurately assessing the risk of antibiotic resistance. To tackle these challenges, additional multicenter randomized controlled trials are recommended [22,23]. In addition, many patients with bronchiectasis also have moderate to severe COPD, and the overlap between these conditions is linked to a worse prognosis than idiopathic bronchiectasis alone. Therefore, there is considerable interest in studying the effectiveness of CFX DPI in bronchiectasis–COPD overlap syndrome (BCOS) [24]. In this article, we thoroughly review various studies to assess the viability of inhaled CFX for LRTIs. We discuss CFX DPI formulations for treating LRTIs in preclinical and clinical settings and highlight the challenges, recent advances, and future directions for managing LRTIs.

### 1.1. Biological Barriers for Effective Inhalation Antimicrobials Delivery

**Figure 2:**
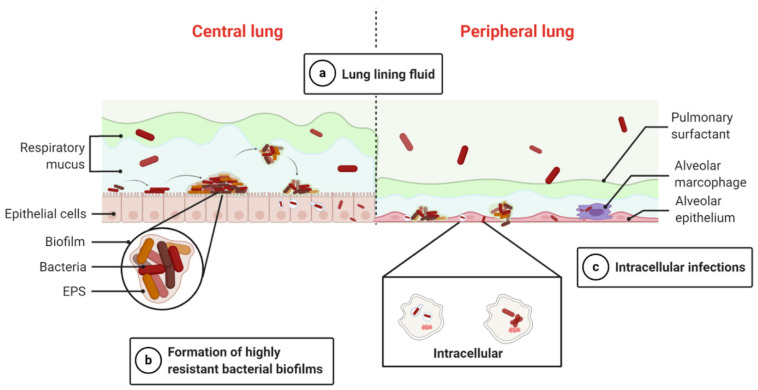
Challenges for efficacious inhalation antimicrobial administration. Adopted from Huang et al. [25].

A range of biological barriers influence the potential administration of drugs to the target site. These barriers exhibit different properties, depending on whether the respiratory tract is in a normal or pathophysiological state. Understanding these biological barriers and the challenges they present is crucial for enhancing drug delivery and, consequently, improving therapeutic outcomes [25]. The biological hurdle for effective antimicrobial administration is demonstrated in Figure 2.

#### 1.1.1. Lung Lining Fluid

Respiratory tract disorders can lead to pathological alterations in the local microenvironment, such as pH and ionic strength. In normal conditions, both the proximal (on the mucus matrix surface) and distal parts of the respiratory tract exhibit a nearly neutral pH in the lung epithelial lining fluid (ELF). Nevertheless, in conditions like CF and COPD, chronic bacterial infections can cause a decrease in pH to 6.0–6.5. This substantial pH shift significantly influences the spatial configuration of mucin molecules, thus impacting the interaction between mucus and nanoparticles [26,27,28,29,30]. Moreover, respiratory tract illnesses trigger excessive mucus production and dehydration, significantly influencing the interaction between the mucus and the administered drug molecules or drug carriers in the lungs. Consequently, these pathological modifications must be considered during the design of effective inhalable nanoparticle carriers. In addition, the pulmonary surfactant, a crucial lipid–protein complex, forms a liquid surface layer on the lung epithelium’s air-liquid interface, consisting of a monolayer and a surface-related reservoir [31]. Besides stabilizing alveoli during breathing, it also plays a vital role in the innate immune defense. Recent research reveals the presence of pulmonary surfactants throughout the respiratory tract, but their composition differs between the central part and the alveoli [32]. The interaction between inhaled nanoparticles and lung surfactants can potentially disrupt biophysical function, leading to nanotoxicity [33].

#### 1.1.2. Bacterial Biofilms

**Figure 3:**
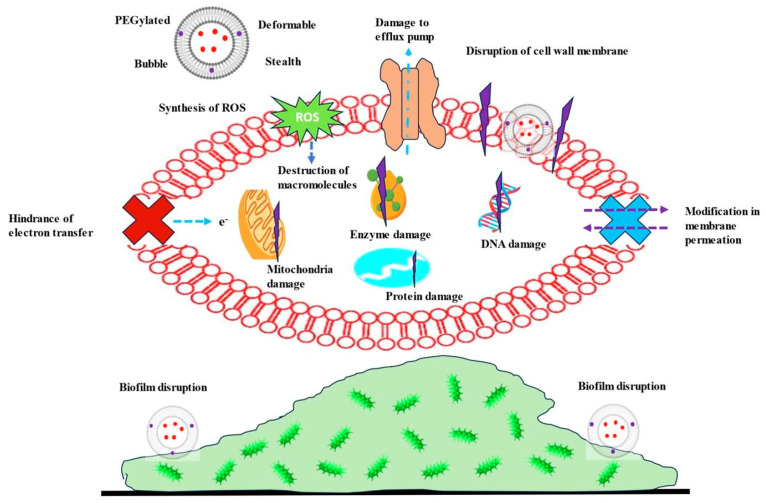
Liposome interaction with bacterial biofilms. Adopted from ref. [37].

Bacterial biofilms are highly organized microbial colonies embedded within a polymeric, carbohydrate-rich extracellular matrix that adheres to either living or inanimate surfaces. The matrix, known as an extracellular polymeric substance (EPS), is composed of proteins, nucleic acids, exopolysaccharides, and other components [34]. Biofilm-related bacterial infections contribute to over 60% of human bacterial infections globally, often resulting in treatment failures in clinical settings [25]. Instances of biofilm infection include *P. aeruginosa* biofilms in CF infections and *Streptococcus pyogenes* biofilms in upper respiratory tract infections. Biofilm formation serves as a safeguarded domain, enabling bacteria to adapt to growth rates and survive in challenging conditions. Biofilm-forming bacteria exhibit remarkable antibiotic resistance, ranging from 100 to 1000 times more than planktonic bacteria [35]. Three proposed mechanisms explain this general resistance: the EPS matrix’s physical barrier, the existence of subpopulations with resistant phenotypes, and the microbial state within the biofilm [34]. The EPS matrix acts as a cohesive, three-dimensional framework that temporarily immobilizes antimicrobials and can deactivate them. Subpopulations in biofilms acquire resistance via several processes, such as preventing drug entry, expelling drugs via active efflux, mutating targets, and enzymatically inactivating drugs. Additionally, the formation of starved, stationary phase dormant regions within biofilms contributes significantly to resistance by limiting the effectiveness of antibiotics requiring cellular activity. Consequently, completely eradicating bacterial biofilms with conventional antimicrobial therapy remains challenging owing to their intricate and various resistance mechanisms [25,36]. Among all nanocarriers, several investigations have revealed that antibiotic-incorporated liposomes are efficacious against bacterial biofilms. When these liposomes interact with bacterial cells, they fuse with the cell membrane and disrupt it. This fusion releases the liposomal contents near the bacteria, which are then absorbed by the bacteria within the biofilm (Figure 3) [37].

#### 1.1.3. Intracellular Infections

Certain bacterial species can infiltrate and persist within host cells in active or dormant states, leading to long-term infections. These bacteria can establish survival niches within host cells, evade immune responses, and induce secondary infections, resulting in persistent or repeated infestations [38,39]. For instance, *Mycobacterium tuberculosis*, when phagocytosed by alveolar macrophages (AMs), can escape engulfing procedures by introducing a survival environment within macrophages or evading into the cytosol [40]. Current epidemiological evaluations highlight the crucial impact of intracellular pathogen, such as *C. pneumoniae* and *M. pneumoniae*, in conditions like asthma, bronchitis, acute pneumonia, CF, and COPD [25]. While most intracellular pathogens infect the mononuclear phagocyte system, various intracellular pathogens can also target nonphagocytic cells like hepatocytes, enterocytes, fibroblasts, and epithelial cells. In addition, some extracellular microorganisms like *S. aureus* and *P. aeruginosa* can penetrate and reside within host cells [41]. Commonly used antimicrobials face challenges of lower intracellular permeation and brief residence duration in the lungs. In overcoming these obstacles, nanoparticle delivery systems must navigate through cellular and intracellular hindrances, entailing efflux pumps, exocytosis, host cell membranes, and endosomal disintegration, to enhance the permeation and deposition of antibacterials within host cells [42].

## 2. CFX DPI: Pharmacokinetics, Pharmacodynamics, and Metabolism

Phase I trials examining the pharmacokinetics (PK) and pharmacodynamics (PD) of CFX DPI have been conducted in healthy individuals and patients diagnosed with CF and COPD. These trials indicated that CFX DPI achieved notably high concentrations within the lungs while maintaining low systemic exposure [17,18,43]. As mentioned earlier, drug concentrations in elevated sputum were markedly more significant compared to those in plasma. For instance, in a study involving CF patients receiving a single dose of CFX DPI (32.5 mg), the drug obtained a C_max_ (mg/L) of 34.9 in sputum and 0.0790 in plasma, with corresponding AUCs (mg/h/L) of 89.5 in sputum and 0.425 in plasma [19]. Additionally, in vivo preclinical studies suggest that CFX DPI exhibited a longer t_1/2_ than soluble CFX hydrochloride, indicating an extended lung residual time. CFX HCl typically demonstrates a serum half-life of around 4 h [44], while in a phase I trial involving healthy volunteers, CFX DPI displayed a terminal half-life of 9.5 h [20]. Data on lung deposition have been collected from healthy individuals as well as patients diagnosed with bronchiectasis, CF, and COPD. Scintigraphic assessments have validated that CFX DPI consistently delivers substantial doses throughout the entire lung, with minimal residual medication left in the device upon delivery [45]. Physiological modeling conducted in healthy volunteers suggested that about 40% of the inhaled dosage reaches the LRT [20]. This percentage is notably higher than the drug retention obtained by other currently available antibiotic DPI products. For instance, Colobreathe^®^ Turbospin^®^ (Forest Laboratories) administered approximately 11.9% and 11.6% to the entire lung, regardless of prior salbutamol treatment [46]. Consequently, CFX DPI holds the potential as one of the most efficient carriers currently available, ensuring extensive distribution across the airways and even reaching the distal airways and alveoli [14].

## 3. Inhalation Devices and CFX Inhalable Formulations: Merits and Demerits in LRTIs

### 3.1. Inhalation Devices

#### 3.1.1. Nebulizers

Nebulizers are devices that produce aerosol droplets ranging between 1 and 5 μm for pulmonary administration. They are especially beneficial for diseases necessitating high pulmonary doses, as they avoid the requirement for drying procedures or propellants. Nebulizers are also helpful for patients who cannot coordinate or achieve the required inspiratory force for inhaling aerosolized medications. There are two main types of nebulizers: jet and ultrasonic, which differ in the method they use to produce aerosols from liquid suspension or solution. Jet nebulizers create aerosol particles through pressure, while ultrasonic nebulizers utilize sound waves to fragment large droplets into smaller ones [47]. Optimization of nebulizers for optimal delivery involves factors such as the air pressure, volume and viscosity of the drug solution, and the type of mouthpiece. Nebulizers are easy to use and can administer large doses of medications mainly in hospitals and clinics under the supervision of an experienced health professional [48].

#### 3.1.2. Metered-Dose Inhaler (MDI)

A metered-dose inhaler (MDI) is a device designed to administer a precise dosage of medication. These inhalers contain a suspension of one or more active ingredients in a propellant, a blend of propellants, or a combination of solvents and propellants. The propellants used in MDI formulations are liquefied gases of chlorofluorocarbon (CFCs), which are not environmentally friendly. To overcome this problem, currently hydrofluoroalkanes (HFAs) that have no effects on the ozone layers are used in the formulations of MDIs. MDI device offers several advantages, including portability and multidose delivery. In mechanically ventilated patients, MDIs are favored over nebulizers due to their overall efficacy [49]. Moreover, MDIs are prevalently employed for the remedy of respiratory tract disorders, such as COPD and asthma. Appropriate coordination between valve actuation and inhalation is essential to deposit into the deep lungs. The inability of many patients to coordinate aerosol actuation and inspiration is very difficult; even after extensive training, many patients cannot operate MDIs appropriately. They can deliver all inhalable drugs, either alone or in combination, in the form of suspensions or solutions. MDIs have been a mainstay of asthma therapy in adults and older children and are now preferred for infants and children under 5 years old, when used with a spacer and, if needed, a well-fitting facemask [48].

#### 3.1.3. Dry Powder Inhaler (DPI)

DPIs are devices utilized to administer powdered drugs to the respiratory tract by using the patient’s inspiratory forces. DPIs offer greater chemical stability because they contain medications in a dried form. However, formulating and manufacturing dried powder particles with the required properties for aerosolization and pulmonary delivery is an intricate process. It is important to note that DPI formulations need to have good flow properties to ensure the dispersion of uniform dosage during inhalation. There are various types of DPI devices (i.e., low-, medium-, and high-resistance), and high-resistance passive DPIs are generally preferable for the patients with limited inspiratory force. The advantages of DPIs over other inhaler systems (i.e., MDIs) are the independence of breathing coordination with dose actuation and the absence of propellants. A DPI is appropriate for those formulations that are targeted to be delivered as powder for inhalation [48]. For instance, Liu and colleagues conducted a study on novel inhalable dry powders of CFX for bronchiectasis therapy. The optimized formulation, containing the highest drug content (80%), showed superior aerosolization efficiency [50].

### 3.2. CFX Inhalable Formulations

#### 3.2.1. Liposomes

Liposomes serve as lipid bilayer structures that encapsulate drugs, functioning as reservoirs to prolong drug release rates and enhance lung exposure periods. They have been widely utilized to load various drugs and treat diverse diseases [51,52]. Liposomal CFX formulations have been created to extend lung residence duration, lessening the need for multiple daily administrations of inhaled antimicrobial therapy. This addresses the challenge posed by the swift absorptive clearance of antibiotics from the lungs [53]. In a comparative evaluation investigating the release of CFX from liposomal and solution formulations designed for inhaled drug administration, the liposomal fabrication exhibited zero-order release kinetics, thereby significantly elevating the drug’s retention time in the lungs [54]. Ong et al. [55] conducted research on the aerosol characteristics of a CFX liposome formulation. Their study revealed that the liposomal preparation effectively managed the controlled release of CFX in cellular models, exhibiting improved antibacterial efficacy against *P. aeruginosa*. This supports the prospective application of inhaled liposomal CFX for treating respiratory infections. Moreover, the fabrication demonstrated a respirable aerosol fraction of 70.5 ± 2.03% of the emitted dose [55]. Mannosylated liposomes carrying CFX were also studied to target intracellular respiratory infections. When administered via the lungs in rats, these liposomes exhibited effective targeting of macrophages [56]. Research performed by Liu et al. [57] developed an optimized CFX liposome with a higher entrapment efficiency of 93.96% and a mean particle size of 349.6 nm (span 0.42), which exhibited prolonged in vitro release. This optimized formulation was then tested in an in vivo investigation using rats, where the proportions of CFX in the lung and blood were measured. The AUC_lung_ value ratio between the CFX liposome and CFX solution was 288.33, indicating a relative bioavailability of 72.42%. Moreover, the drug-targeting effectiveness of the CFX liposome by intratracheal delivery was markedly superior to the CFX solution, with values of 799.71 and 2.01, respectively [57]. Additionally, research conducted by Zhang’s team prepared CFX liposomes and their study showed −10 mV and 105 nm for surface charge and particle size, respectively. Slower in vitro drug release was observed up to 24 h (95%) [58]. As with previous clinical trials, administering liposomal CFX via inhalation resulted in a decrease in bacterial burden. Although there were no noticeable enhancements in lung function, the therapy was well-suited, with comparable rates of detrimental reactions in both study groups [59]. Considering the recent trial results, inhaled CFX has correlated with reduced bacterial load and positive clinical outcomes. Consequently, inhaled liposomal CFX has emerged as an appealing treatment choice for individuals with bronchiectasis and chronic *P. aeruginosa* infection [60]. Researchers have highlighted the suitability of liposomal formulations in targeting the alveolar epithelial cell lining fluid, which is crucial for treating deep lung diseases. However, most inhaled liposomal fabrications are typically delivered via nebulization due to their liquid form and face challenges related to colloidal stability and potential drug leakage during this process, limiting their clinical applications [61,62].

#### 3.2.2. Micelles

A micelle is an aggregate of surfactant molecules, which have both hydrophilic and hydrophobic parts, assembled into a colloidal suspension in a liquid [63]. It has been reported that copolymer micelles demonstrate a slow degradation rate similar to that of polymeric nanoparticles, potentially harming lung tissues. For instance, treating *P. aeruginosa*-infected mice with micellar CFX exhibited controlled release of CFX as needed, resulting in decreased bacterial presence and reduced alveolar damage [64]. A notable benefit is that antimicrobial agents based on polymeric micelles do not trigger bacterial resistance [65,66]. Cationic polymeric micelles commonly feature quaternary ammonium or tertiary amino groups. Micellar systems utilizing materials such as chitosan, poly[2-(*tert*-butylaminoethyl) methacrylate], poly(amidoamine), and others have demonstrated effective antibacterial characteristics over various bacterial strains [67,68]. However, it is noteworthy that polycations are often linked with notable cytotoxicity, mainly while delivered at higher doses [69]. In a recent study, Stancheva and colleagues [65] developed CFX-encapsulated mixed polymeric micelles (MPMs) as agents to combat biofilms. They assessed several physicochemical characteristics of MPMs, such as size, size distribution, and critical micellar concentration (CMC). The resulting MPMs were tiny, with a hydrodynamic diameter of approximately 35 nm. The surface charge and CMC values of the MPMs were closely linked to their composition. The study found that between 50% to 80% of the drug was released within the initial 4 h, depending on the composition of the micelles. All micellar systems effectively detached pre-formed bacterial biofilms of *E. coli* and *S. aureus*, considerably reducing their biomass. The CFX-loaded MPMs successfully suppressed the metabolic activity of the biofilms, demonstrating effective drug delivery and release. Farhangi et al. [70] conducted a study to optimize a dry powder inhaler (DPI) containing CFX-incorporated polymeric nanomicelles using a spray drying technique. The nanoaggregates decomposed into nanomicelles with a mean particle size of 291.1 nm and a polydispersity index of 0.214. Importantly, the spray drying process did not adversely affect the stability or drug release profile of the nanomicelles. Furthermore, the antibacterial efficacy of CFX against *Klebsiella pneumoniae*, *P. aeruginosa*, and *Streptococcus pneumoniae* was significantly enhanced [70].

#### 3.2.3. Nanosuspensions

The nanosuspension mode of pulmonary delivery has the potential to penetrate deeply into the lungs and reach smaller airways, promoting a more homogeneous dispersion of the drug. This can enhance the accuracy of drug distribution modeling and ultimately improve efficacy [71]. Recently, Liu and colleagues developed a dry powder formulation containing nanosuspensions of CFX and curcumin to remedy lung infestations. The study demonstrated that this multidrug powder exhibited potent antibacterial effectiveness even at low doses, effectively alleviating the systemic toxicity associated with high-dose administration. In vitro experiments assessing drug release revealed excellent release properties for the powder. Consequently, the authors concluded that their co-delivery system, which integrates curcumin nanosuspensions and CFX with *N*-acetylcysteine dry powder, proved well-suited for treating lung infections [72]. In conventional suspension aerosols, many droplets lack drug content, while others contain high drug concentrations, leading to uneven drug release and distribution within the lungs [73].

#### 3.2.4. Chitosan Loaded Nanoparticles

Chitosan is frequently used to encapsulate antibiotics for nano-delivery [74]. The effectiveness of chitosan as a carrier for pulmonary particulate drugs depends on its mucoadhesive properties, ability to enhance biofilm permeation, and specific targeting to sites or cells. For nanocarriers, different systems like microencapsulation and micro–nano blending have been developed to give them suitable aerodynamic properties for efficacious pulmonary aerosolization and inhalation [75,76,77]. Moreover, chitosan nanocarriers, including solid nanoparticles and liquid nanoemulsions, have been studied for their potential to deliver anti-tubercular drugs to the lungs [78]. Nanoemulsions, delivered through pulmonary nebulization, disperse well and can reach the peripheral lungs efficiently. Decorating nanoemulsion droplets with chitosan and folate in a covalent conjugate form enhances particle endocytosis into macrophages and improves lung drug retention [78,79,80]. Vildan and colleagues conducted a study on the in vitro antimicrobial effectiveness of CFX-incorporated chitosan microparticles and their impacts on human lung epithelial cells (BEAS-2B). The study found that only the CFX–chitosan microparticles, not the chitosan microparticles alone, hindered bacterial growth at non-cytotoxic concentrations to BEAS-2B. The CFX–chitosan microparticles damaged the bacterial cell wall and membrane, and those ≤200 nm in size were absorbed by both BEAS-2B cells and pathogens [81]. In addition, research has shown that CFX-loaded chitosan nanoparticles have a 50% lower minimum inhibitory concentration (MIC) compared to CFX alone against *S. aureus* and *E. coli*. This loading approach enhanced the permeation of the drug into bacterial cells, ameliorating antibacterial action and allowing for a reduced dose [82]. Another study reported that CFX exhibited its most vital antibacterial effectiveness against MRSA and *E. coli* when loaded in chitosan, resulting in an 85.6% decline in MIC. This approach also improved drug administration, elevated drug permeability, and provided prolonged release [83]. CFX chitosan microparticles designed for targeted drug administration to the lungs against pathogens that cause pneumonia (*S. aureus*, *P. aeruginosa*, and *E. coli*) showed better loading potentiality. They also showed elevated localized efficaciousness, better evasion of the body’s local defense system, enhanced antimicrobial effectiveness, and minimal toxicity to human lung epithelial cells [81]. However, chitosan nanoparticles tend to aggregate and be exhaled when inhaled into the lungs. Blending nanoparticles with lactose microparticles of approximately 5 µm in size could reduce their tendency to clump together through a surface adsorption phenomenon [84]. Egorov and colleagues developed chitosan-based self-assembled nanoparticles for delivering CFX. Their study showed that these systems have high loading and encapsulation efficiency, with an extended-release profile lasting up to 20 h [85].

#### 3.2.5. Solid Lipid Nanoparticles

Stearic acid-containing solid lipid nanoparticles exhibited a pronounced burst effect and rapid release of CFX [86]. To address the biological (mucus) hurdle, researchers incorporated CFX into lipid-core nanocapsules (LNC) to enhance mucus penetration, sustain release, and maintain antimicrobial action. This approach led to a 50% enhancement in drug permeability through mucus. The study highlighted several benefits, including high entrapment capacity, elevated aqueous solubility, depleted frequency of dosing and total dose, prevention of biofilm formation, enhanced mucus penetration, and shielding of the drug from inactivation. These advantages collectively suggest that CFX-loaded LNC could be a potential drug carrier for enhancing antibiotic treatment in LRTIs [87]. However, due to their highly crystalline structure, solid lipid nanoparticles exhibit low efficiency in loading drugs [88].

#### 3.2.6. Metal-Containing Nanoparticles

The remedy of persistent lung infections presents a significant challenge owing to drug-resistant bacteria. Developing a novel drug based on lipid–metal conjugation encapsulated with potent antibiotics offers improved drug delivery. In a study by Liu et al., CFX-loaded selenium–lipid nanoparticles (CFX-LSENPs) were produced using a new method, and their antimicrobial effectiveness was assessed against the clinically important Gram-negative bacteria *P. aeruginosa*. The CFX-LSENPs exhibited superior antibacterial efficacy on *P. aeruginosa*, leading to a drastic decline in bacterial count compared to the control group. The number of dead bacterial cells was markedly greater in the CFX-LSENP-treated groups. Additionally, CFX-LSENPs increased the activities of antioxidant enzymes (lipid peroxidation, catalase, superoxide dismutase, and glutathione peroxidase) in mice and protected against liver damage from bacterial infection. The research suggests that these developed nanoparticles could be used as potent antimicrobial and antioxidant agents for managing lung infections or interstitial lung ailments [89].

### 3.3. Commercialized Formulations of Inhaled CFX

Two types of liposomal CFX formulations are available commercially: rapid-release formulations (Lipoquin™ or ARD-3100) and slow-release formulations (Pulmaquin™ or ARD-3150). Lipoquin™ contains CFX in a liposomal formulation, while Pulmaquin™ is a dual-release CFX for inhalation (DRCFI) that mixes Lipoquin™ with a solution of free CFX to facilitate an initially high peak of CFX in the lung. In a phase II clinical trial with 22 adult CF patients, the safety and tolerability of Lipoquin™ were demonstrated with once daily (OD) administration of a 300 mg dose over 14 days [90,91]. The excellent tolerability in CF patients led to the investigation of Lipoquin™ in bronchiectasis sufferers colonized with *P. aeruginosa*, a group historically unresponsive to inhaled antibiotics, unlike CF patients [92]. Further phase II clinical trials (ORBIT-1 for Lipoquin™ and ORBIT-2 for Pulmaquin™) assessed the effectiveness and safety of liposomal CFX in adults with chronic *P. aeruginosa* lung infection caused by CFX-sensitive strains and CF/bronchiectasis. These trials showed that an OD inhaled dose had potential antimicrobial effects against *P. aeruginosa*, was well-suited, and diminished the risk of *P. aeruginosa* exacerbation [91,93]. Based on all available preclinical and clinical data, a 6 mL OD dose of Pulmaquin™ was chosen for the phase III program in bronchiectasis, including the ORBIT-3 and ORBIT-4 trials. Although Pulmaquin™ remarkably extended the period to initial pulmonary exacerbation in ORBIT-4 than placebo, this effect was not seen in ORBIT-3 or the combined analysis. The inconsistencies between trials and the underwhelming findings indicate the necessity for further investigation but also suggest a potential rationale for using inhaled CFX in this specific context [94].

### 3.4. Potential Biomarkers for Identifying CFX Resistance in LRTIs

Biomarkers are measurable indicators that establish a connection with a disease or physiological condition, offering support for diagnostic or predictive assessments of specific pathologies. The reliability of a biomarker is contingent on factors such as accessibility, sensitivity, and specificity. In the context of CF, pulmonary biomarkers are increasingly employed to examine disease action and assess the response to therapy in individuals [95]. Recently, Su et al. [96] conducted research indicating that an increased biosynthesis of fatty acids plays a crucial role in CFX resistance development in *P. aeruginosa*. Their study revealed that the elevated fatty acid formation is a distinctive property of metabolomes resistant to CFX, augmented by heightened gene expression and enzymatic action within the biochemical network. The use of the fatty acid synthase inhibitor triclosan enhanced the effectiveness of CFX in eliminating PA-R128CIP and clinically multidrug-resistant *P. aeruginosa* strains. This enhanced efficacy was accompanied by declined molecular transcription and enzymatic process, leading to the restoration of normal fatty acid levels in the metabolic cascade. These findings underscore the significance of increased fatty acid biosynthesis in the CFX resistance of *P. aeruginosa*, offering a potential target cascade for addressing CFX-resistant strains of the bacteria. The discovery of novel biomarkers is inevitable for enhancing the remedy of CF in patients [96].

### 3.5. CFX Inhalable Formulations for LRTIs Management in Preclinical Settings

To date, researchers have achieved mounting success on CFX inhalable formulations for potentially managing LRTIs in experimental settings. Research performed by Yu and the team prepared inhaled liposomal powder fabrications for co-administration of CFX and colistin and examined their synergistic efficacy against multidrug-resistant Gram-negative lung infestations. In their study, cytotoxicity evaluation revealed that the optimized liposomal preparation was non-cytotoxic at the drug concentration of 5 µg/mL and 20 µg/mL for colistin and CFX, respectively. Colistin monotherapy (2 mg/L) has shown no antibacterial effectiveness against *P. aeruginosa* H133880624 and H131300444. Similarly, CFX (8 mg/L) monotherapy revealed reasonable bacterial killing for both clinical isolates; however, regrowth appeared in 6 h for *P. aeruginosa* H133880624. The combinational liposomal fabrication showed considerable antibacterial efficacy against both clinical isolates of *P. aeruginosa* compared to each antibiotic [97]. Apart from this, Chai et al. [98] investigated combining colistin and CFX within liposomes utilizing an in vitro human lung epithelial cell model. Their findings revealed that delivering both drugs in a single liposome reduced their transportation capacity to cross the lung epithelial cell layer while elevating their deposition on the lung epithelial surfaces. Consequently, this strategy holds promise for managing pulmonary ailments triggered by multidrug-resistant *P. aeruginosa* [98].

**Figure 4:**
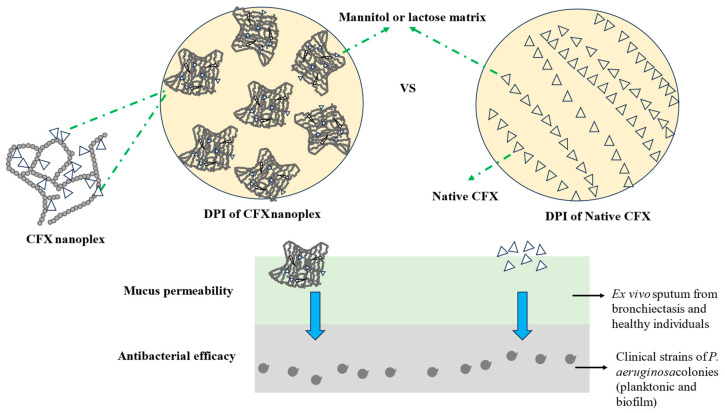
DPI formulation of CFX nanoplex exhibits better mucus permeation and antibacterial effectiveness compared to the native CFX.

Furthermore, a study by Almurshedi and coworkers developed CFX-loaded nanostructured lipid carriers and examined their effectiveness against bronchiectasis. Their study showed a remarkable improvement in the fine particle fraction (FPF) with enhancing lipid: chitosan proportions. Their findings concluded that novel inhalable CFX-loaded nanocomposite microparticle powders are a promising new strategy to ameliorate target potentiality and administration of CFX for bronchiectasis therapy [99]. In addition, Hisham and colleagues fabricated inhaled hybrid silver/CFX nanoparticles with synergistic effectiveness against *P. aeruginosa*. In their research, in vitro deposition findings demonstrated notable accumulation in stage 2 using twin-stage impinger (~70%). In comparison with CFX, the prepared hybrid nanoparticles were 3–4-fold more efficacious against impeding growth and biofilm formation by *P. aeruginosa* PA01 and *P. aeruginosa* NCTC 10662 [100]. Furthermore, Tran and the team prepared and assessed inhalable CFX nanoplex in conjunction with mannitol as an innovative bronchiectasis treatment. In this study, a CFX nanoplex was non-toxic to cells and similar to CFX native against 16HBE14o- and A549 cell lines (≈70–80% cell survival at 0.1 mg/mL). The antibacterial effectiveness against *P. aeruginosa* was markedly higher with a CFX nanoplex in comparison with CFX native (Figure 4). The authors reported that their new CFX nanoplex illustrated amelioration in a CFX DPI as a bronchiectasis therapeutic [101].

Moreover, another study by Tran and colleagues examined the DPI formulation of a CFX nanoplex with mannitol/lactose as the excipient for bronchiectasis treatment. Despite achieving similar rapid dissolution rates in sputum showed by CFX nanoplex and CFX native, the dry powder inhaler of CFX nanoplex revealed markedly higher mucus permeation than CFX native (5–7-fold increase) assigned to its built-in capacity to yield significantly supersaturated CFX levels in the sputum. The higher mucus permeation resulted in a greater antibacterial effectiveness of the CFX nanoplex (>3 log10 CFU/mL). The cytotoxicity of the CFX nanoplex, in terms of DPI, was similar to that of the native CFX, suggesting a minimal likelihood of toxicity when applied to lung epithelial cells. These findings introduced the optimistic ability of DPI of CFX nanoplex as a novel remedial approach for bronchiectasis [102]. In addition, a study performed by Tureli et al. fabricated CFX-incorporated PLGA nanoparticles and examined their antibacterial efficacy against *P. aeruginosa* in lung infections associated with CF. In their research, the cytotoxicity assessments conducted on Calu-3 cells and CF bronchial epithelial cells (CFBE41o-) demonstrated that the PLGA nanoparticles encapsulated with the complex exhibited no toxicity at concentrations considerably greater than the MIC against laboratory strains of the bacteria. Furthermore, tests evaluating their antibacterial properties revealed improved effectiveness when administered in nanoparticle form. The colloidal stability of these nanoparticles within mucus was also confirmed. Importantly, a noticeable reduction in mucus was observed when the nanoparticles were incubated with mucus turbidity. Therefore, these CFX complex-incorporated PLGA nanoparticles are now established as potential nano drug carriers for combating *P. aeruginosa* infestations in the lungs of CF patients [103]. Hamblin and colleagues evaluated inhaled liposomal CFX as a potential therapy for infection with *Yersinia pestis*. A single regimen of CFI, but not DRCFI, exhibited a notable enhancement in survival compared to a single dosage of CFX. Additionally, both CFI and DRCFI in single doses effectively diminished the bacterial density in the lungs and spleen to levels undetectable at 60 h following the challenge. However, when treatment initiation was postponed until 42 h post-challenge, a sole dose of CFI or DRCFI provided only lesser protective effects. Nevertheless, single doses of CFI or DRCFI managed to diminish the microbial load in the spleen markedly more than blank liposomes. A three-day therapeutic course involving CFX, CFI, or DRCFI led to remarkably elevated shielding, with survival rates ranging from 90% to 100%. This finding implies that CFI and DRCFI may be valuable therapeutic options for *Y. pestis* infection, preventive measures, and treating plague [104].

**Figure 5:**
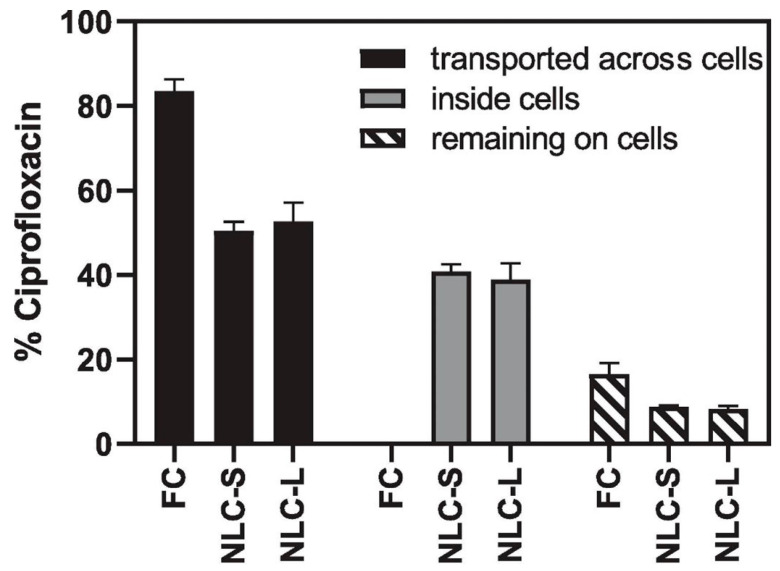
Distribution of CFX intracellularly, residual on the H411 epithelial cells and carried through these cells after 4 h for free CFX, and nano-liposomal CFX. FC: free CFX, NLC-S: nano-liposomal CFX with small vesicle, NLC-L: nano-liposomal CFX with large vesicle. Adopted from ref. [58].

Furthermore, Zhang et al. [58] recently evaluated the antibiofilm effectiveness of two inhalable liposomal CFX fabrications with varied vesicle sizes fabricated by applying a 3D-printed microfluidic chip. Their findings indicated that both liposomal-contained CFX preparations and the free CFX solution exhibited similar properties in terms of aerosolization and effectiveness in eradicating biofilms. Notably, the CFX liposomal formulation with a smaller vesicle size exhibited a considerably slower release of the drug when subjected to the dialysis bag approach in comparison with the free CFX solution. Surprisingly, CFX liposomal fabrications effectively controlled drug release when tested in the alveolar epithelial H441 cell model, and they exhibited unique drug transport behaviors in H441 cell lines when contrasted with the free CFX solution (Figure 5), indicating the potential of inhaled liposomal CFX as a promising remedial strategy for respiratory infections [58].

**Figure 6:**
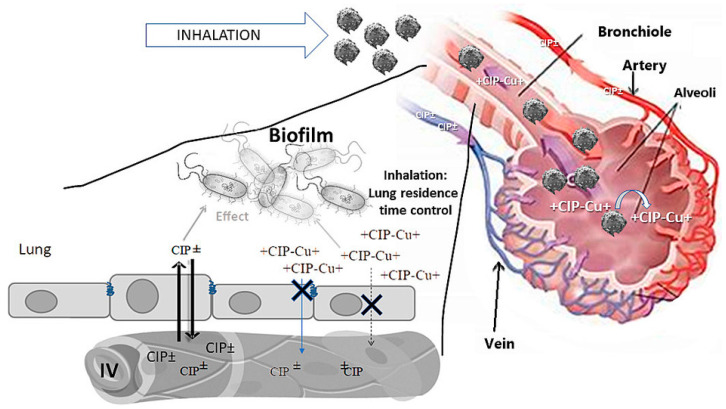
Inhaled microparticles incorporated with the CIP-Cu^2+^ complex revealed higher pulmonary exposure than IV solution. CIP: ciprofloxacin. Adopted from Tewes et al. [105].

A study conducted by Liu’s team developed novel inhalable CFX dry powders for bronchiectasis therapy. The optimized formulation, with the highest drug content (80%), demonstrated superior efficiency in terms of aerosolization. It achieved an FPF of 45.04  ±  0.84%, an emitted dose of 98.10  ±  1.27%, and a mass median aerodynamic (MMA) diameter of 3.75  ±  0.03 μm. The increased drug content was achieved through electrostatic interactions between silk fibroin (SF) and CFX via adsorption. The improved aerosolization performance can be mainly associated with SF’s soft and airy texture nature and light-density configuration. These findings suggest that the novel inhalable microparticles containing CFX, based on silk, could offer a promising approach to treating bronchiectasis [50]. Tewes’s team performed research comparing the PK and effectiveness of microparticles incorporated with a CFX-Cu^2+^ complex, administered via the pulmonary route, with an IV solution of CFX in a rat model with persistent lung ailment. Following a single pulmonary delivery of CFX-Cu^2+^ complex-incorporated microparticles, the pulmonary exposure to CFX increased dramatically, by 2077-fold, compared to the IV delivery of a CFX solution. This singular lung delivery resulted in a remarkable 10-fold decline in the lung burden of *P. aeruginosa*, as evaluated by colony-forming units (CFU) per lung, 24 h after administration, whereas IV administration of the same CFX dose was inefficacious compared with the untreated control group. The superior effectiveness of inhaled microparticles incorporated with the CFX-Cu^2+^ complex, as opposed to a CFX solution, can be linked to the substantially superior pulmonary exposure to CFX achieved through the inhalation of CFX-Cu^2+^ complex-loaded microparticles compared to the IV solution (Figure 6) [105].

**Figure 7:**
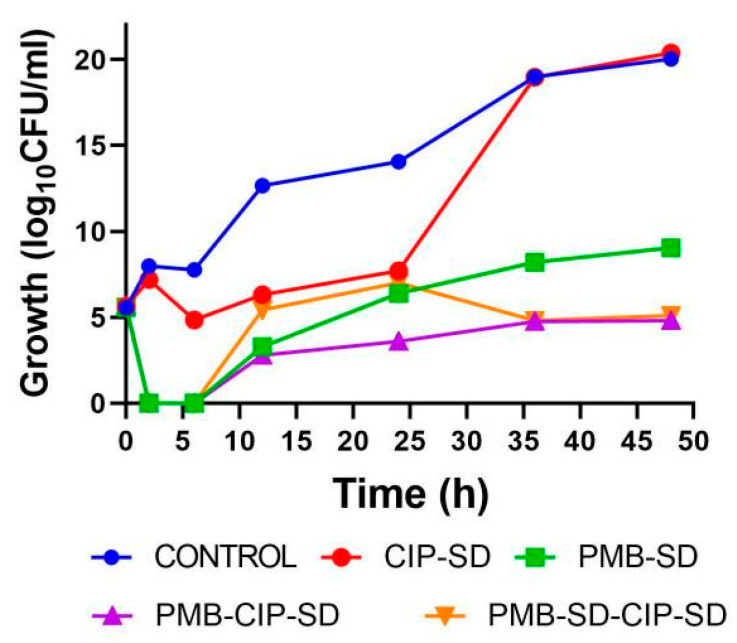
Time-kill experiment with *A. baumannii* K31 treated with various formulations. Control: no antibiotic, CIP-SD: ciprofloxacin spray-dried powder, PMB-SD: polymyxin B spray-dried powder, PMB-CIP-SD: co-spray-dried powder of PMB and CIP, PMB-SD-CIP-SD: PMB spray-dried powder and CIP spray-dried powder. Adopted from Wang et al. [111].

In addition, Tewes et al. [106] prepared inhalable calcium-mediated inorganic-organic composite microparticles to prolong the presence of CFX in the lungs. In their research, CFX was entirely discharged from the microparticles within 7 h, displaying dissolution patterns that exhibited slight reliance on pH levels (at both pH 5 and 7.4) compared to pure CFX. Investigations involving the transportation of CFX across Calu-3 cell monolayers, while varying calcium levels, revealed an 84% reduction in CFX apparent permeability. Interestingly, the apparent MIC of CFX against *P. aeruginosa* and *S. aureus* remained unaltered in the presence of identical calcium concentrations. These outcomes suggest that the developed particles hold promise in maintaining sustained CFX levels in the lungs to achieve therapeutic effects. With these microparticles, regulating CFX PK within the lung becomes plausible, achieved through controlled CFX release from the particles and diminished apparent permeation across the epithelial hindrance due to interactions between the cation and CFX [106]. Additionally, research performed by Shi et al. [6] assessed lung exposure of several inhalable CFX formulations with various release rates in a rat model. In their research, CFX spray-dried powder (CHDP) demonstrated the most rapid drug release rate, whereas the CFX microcrystalline dry powder (CMDP) and CFX nanocrystalline dry powder (CNDP) showed considerably slower drug release. Furthermore, CMDP and CNDP showed markedly higher in vivo lung exposure to CFX in comparison to both CHDP- and CFX-loaded PLGA micro-particles (CHPMs). This finding implies that the lung exposure to inhaled medications with high permeation is influenced by the drug release rate, suggesting that enhancing lung exposure to inhaled antibiotics could be achieved through a prolonged-release fabrication approach [6]. In addition, Sabuj et al. [1] evaluated the fabrication of CFX-encapsulated poly(2-ethyl-2-oxazoline) (PEtOx) nanoparticles for efficient pulmonary administration from DPI preparations against LRTIs. The in vitro aerosolization investigation demonstrated an FPF ranging from 34.4% to 40.8%. The FPF elevated proportionally with higher drug loading. These results highlight the promise of utilizing the polymer PEtOx as a carrier for developing CFX-loaded PEtOx nanoparticles in DPI preparations for the management of LRTIs [1]. Further, the study carried out by Lin and team prepared inhaled combinational powder fabrications of phage and CFX and examined its antibacterial efficacy against *P. aeruginosa*-associated respiratory infections. In their study, both formulations A and B demonstrated excellent synergistic antimicrobial killing efficacy of the two *P. aeruginosa* strains (FADD1-PA001 and JIP865). Both fabrications preserved bactericidal synergy following dispersion through lesser and higher resistance osmohaler^TM^. As per these findings, the authors have concluded that it is achievable to fabricate stable and inhalable combinational powder fabrications of phage PEV20 and CFX for the effective management of respiratory infestations associated with multidrug-resistant (MDR) *P. aeruginosa* [107]. Arauzo et al. [108] prepared dry powder preparations of CFX loaded in chitosan sub-micron particles for pulmonary infections. In their study, particles revealed biocompatibility with the alveolar cell line (A549) and demonstrated an efficacious antimicrobial action against two of the most prevalent respiratory pathogens, including *P. aeruginosa* and *S. aureus* [108]. Furthermore, Karimi and colleagues developed a microparticle-based DPI formulation of CFX that applied a quality-by-design strategy. In their study, formulations exhibited an adequate size within the 2–4 µm range and revealed an ameliorated aerosol efficiency with FPF up to 80% [109]. Recently, a study conducted by Xu and his team developed inhalable CFX/polymyxin B (PMB) dry powders for managing respiratory infections. These powders also maintained their ability to combat *P. aeruginosa* strain PAO1. Furthermore, when kept at 3% relative humidity and a temperature of 20 ± 5 °C for 4 months, the spray-dried powder formulations showed good stability in their solid-state form and suitable aerodynamic properties. Overall, inhalable dry powders containing CFX and PMB are promising treatments for respiratory tract infections [110]. In addition, Wang and colleagues developed inhalable dry powder formulations containing a combination of CFX and PMB. The co-administration of CFX and PMB demonstrated synergistic antibacterial effectiveness against *A. baumannii* and inhibited the emergence of resistance to these drugs. Genomic analysis revealed only minor genetic variations between the mutants and the original strain, consisting of three to six single nucleotide polymorphisms (SNPs). This research indicates that inhalable spray-dried powders comprising CFX and PMB hold promise for treating respiratory infections caused by *A. baumannii*, offering improved bacterial eradication and resistance prevention. In their study, co-spray-dried powder of PMB and CFX demonstrated the strongest bacterial inhibition effect (Figure 7) [111].

Liu and colleagues [72] prepared a nanosuspension-based DPI formulation of CFX and curcumin to manage lung infections. The research showed that the multidrug powder exhibited effective antibacterial efficaciousness even at low doses, consequently minimizing the systemic toxicity associated with high-dose administration. In vitro experiments examining drug release also indicated excellent release properties for the powder. As a result, the authors concluded that their codelivery system, which combines curcumin nanosuspensions and CFX with *N*-acetylcysteine dry powder, was well-suited for treating lung infections [72]. Chono and colleagues [112] performed research examining the efficacy of delivering liposomal CFX through pulmonary administration in treating pneumonia. The research examined the PK of liposomal CFX in rats with lipopolysaccharide-enhanced pneumonia. The study found that after pulmonary administration of liposomal CFX, the concentration of CFX in both AMs and lung ELF followed significantly higher time courses than the free CFX administration at the same dosage (200 mcg/kg). Conversely, the concentration of CFX in the plasma following pulmonary delivery of CFX liposomes was notably less than that in AMs and ELF. These findings reveal that pulmonary delivery of liposomal CPFX was more efficient in administering CFX to AMs and ELF than free CFX. Additionally, it prevented CFX dispersion into the bloodstream. As per PK/PD assessment, liposomal CFX demonstrated potent antibacterial effectiveness against pneumonia-causing pathogens. Consequently, this study advises that CFX pulmonary delivery could potentially treat pneumonia [112]. Shetty et al. [113] conducted a study where they developed combined formulations for dry powder inhalers by co-spray drying colistin and CFX in mass proportions of 1:1, 1:3, and 1:9. Their investigation revealed that co-spray drying CFX with colistin in a 1:1 mass ratio effectively hindered the crystallization of CFX for up to 60 days at 55% relative humidity (RH). However, the formulation containing colistin and CFX in a 1:1 ratio experienced fusion when exposed to 75% RH during storage, leading to compromised aerosol performance due to moisture absorption. Conversely, the formulation comprising colistin, CFX, and leucine in equal proportions (1:1:1) exhibited no particle fusion, ensuring stable aerosol efficiency for 7 days at 75% RH [113]. Furthermore, Yagmur et al. [114] developed DPIs containing CFX or levofloxacin along with the mucolytics acetylcysteine and dornase alfa, intended for treating lung infections in CF patients. Their research revealed that the release profiles of micro-homogenized and spray-dried CFX were superior to those of free CFX. Additionally, the findings obtained from the Andersen cascade impactor (ACI) demonstrated that all fabrications had MMA diameters below 5 μm [114]. Research conducted by Nasser and team [115] assessed the potentiality of creating inhalable particles containing a combination of CFX and quercetin in a co-amorphous form, aiming to enhance the stability of the amorphous state compared to the individual drugs. The study found that spray-dried combinations of CFX–quercetin (1:1 M ratio) led to co-amorphous systems that showed increased stability and better aerosol efficiency [115]. The comprehensive overview of preclinical study data is shown in Table 1.

### 3.6. CFX Inhalable Formulations for LRTI Management in Clinical Settings

Various CFX-loaded inhalable fabrications have recently demonstrated beneficial outcomes against LRTIs. A study conducted by Wilson’s team performed a phase II, randomized, double-blind, multicenter research to examine the safety and effectiveness of CFX DPI in bronchiectasis patients. In their study, CFX DPI (given at 32.5 mg twice daily for 28 days) was well-suited and showed remarkable decreases in overall bacterial burden compared to the placebo in subjects with bronchiectasis [23]. In addition, Stass and colleagues carried out a phase I, single-dose, randomized, placebo-controlled study in a hospital setting where subject follow-up was performed for 2 weeks for safety. In this study, the findings revealed that CFX DPI was well-tolerated with no clinically associated detrimental impacts on lung function. According to PK models based on physiological factors, it was suggested that approximately 40% of the entire CFX DPI dose reached the trachea, bronchi, and alveolar space. CFX in the systemic circulation was observed shortly upon inhalation, but the overall systemic exposure was minimal. Data on the terminal elimination half-life, apparent overall clearance from plasma following non-intravenous delivery, and apparent volume of distribution all indicated that eradication from the respiratory tract was extended, reporting further evaluation of its clinical effectiveness for the treatment of specified, persistent infestations in lung ailments [20]. Additionally, Stass and colleagues performed a phase I, randomized, single-dose, dose-escalation evaluation and examined the safety and PK of CFX DPI in CF. The administration of single doses of CFX DPI at either 32.5 mg or 65 mg was well-received, with comparable rates of detrimental effects observed across all participant groups. There were no instances of mortality, therapy discontinuations, critical harmful effects related to treatment, or notable alterations in laboratory parameters, vital signs, or pulmonary function assessments. In conclusion, the study affirmed the effective targeting of the lungs, achieving high concentrations of CFX in pulmonary regions while minimizing systemic exposure. These findings provide valuable support for further exploration of CFX DPI as a possibly more advantageous substitute to nebulized antibiotic solutions for managing persistent lung infestations [19]. Besides, Stass and team again performed a phase I randomized research and assessed tolerability and PK characteristics of CFX DPI in CF sufferers. CFX DPI was quickly absorbed upon inhalation without deleterious severe effects related to treatment. Systemic exposure to CFX remained low and was similar for both single and multiple doses across the three dose groups, indicating a lack of significant drug deposition in the body [18]. Additionally, Stass and colleagues performed a phase I clinical trial with a randomization, single-blind approach. Their research focused on examining the safety and PK of several dosages of CFX DPI in individuals with moderate to severe COPD. Importantly, there was no serious adverse event occurrence; instead, the most detrimental reactions were mild in nature. The study’s primary finding was that CFX DPI was well-tolerated when given to patients with moderate or severe COPD for 12 days. Interestingly, they noted that the drug attained elevated levels in sputum while resulting in minimal systemic exposure [17].

Moreover, in another investigation by Haworth and team, two phase III randomized controlled trials were conducted involving inhaled liposomal CFX (ARD-3150) in patients with bronchiectasis and recurrent lung infestation caused by *P. aeruginosa*. The study revealed that in the ORBIT-4 trial, participants in the ARD-3150 had a median time of 230 days before experiencing their first pulmonary exacerbation, whereas the placebo group had a median duration of 158 days before the initial exacerbation. This 72-day difference between the two groups was statistically notable, although it was not observed in ORBIT-3 or the combined assessment. This finding also emphasized that the discrepancies obtained between the trials underscore the need for additional assessment of the heterogeneity within bronchiectasis. It also highlighted the importance of identifying the most appropriate benchmark for assessing the efficaciousness of inhaled antibiotics [94]. Furthermore, research conducted by Serisier and his team employed liposomal CFX in bronchiectasis. This phase II trial, spanning 24 weeks and entailing various sites in Australia and New Zealand, recruited 42 adults diagnosed with bronchiectasis. These individuals had suffered a minimum of two pulmonary exacerbations in the last year and were identified as having *P. aeruginosa* bacteria responsive to CFX during screening. The trial followed a randomized, double-blind, and placebo-controlled approach. In their investigation, the use of DRCFI led to a decrease in *P. aeruginosa* burden by day 28. Additionally, DRCFI therapy prolonged the duration until the initial pulmonary exacerbation (median 134 days vs. 58 days) and was better tolerated, exhibiting a similar incidence of systemic unintended effects compared to the placebo group but lesser deleterious impacts on the lungs [91]. Dorkin and team conducted a placebo-controlled, randomized, phase IIb clinical trial involving 288 adolescent and adult patients with CF. These patients were divided into groups receiving either a placebo or CFX DPI at 32.5 mg or 48.75 mg doses, administered twice daily for 29 days. The study did not reveal any considerable variations in the primary efficacy endpoint, which measured the alteration in forced expiratory volume in 1 s (FEV1), between the CFX DPI groups and their respective placebo groups, regardless of the dosage. However, when the data from both dosages were combined and analyzed together, CFX DPI was related to a statistically notable decline in the FEV1 compared to the placebo. Additionally, CFX DPI demonstrated beneficial outcomes on sputum bacterial burden and well-being. It is worth noting that these positive outcomes were not extended at the 4-week follow-up. As a result, the authors recommended further investigations to fully comprehend the extent of the advantageous impact of CFX DPI for patients with CF [116]. Apart from this, a study conducted by Chalmers and colleagues investigated modifications in respiratory symptoms over a 48-week treatment period with ARD-3150 in bronchiectasis. Their study demonstrated that ARD-3150 therapy resulted in notable enhancements in respiratory symptoms while patients were actively undergoing therapy. However, these ameliorations waned during treatment-free intervals [117].

Additionally, Anthony and colleagues assessed the safety and effectiveness of CFX DPI in bronchiectasis patients with a phase III, double-blind, placebo-controlled trial. Patients were unpredictably allocated in a 2:1 ratio to receive either twice-daily CFX DPI at a dose of 32.5 mg or a placebo. These treatments were delivered in two different regimens, involving cycles of 14 or 28 days, and the total treatment duration was 48 weeks. The study’s primary endpoints included measuring the time it took for the initial exacerbation to occur and assessing the incidence of exacerbations. The results showed that the use of CFX DPI in a 14-day on/off treatment cycle extensively extended the time until the first exacerbation compared to a pooled placebo, and it also diminished the recurrence of exacerbations by 39% compared to the placebo group. However, the outcomes for CFX DPI in the 28-day on/off regimen did not show statistically significant differences from the placebo. In terms of safety, the profile of CFX DPI was favorable. Based on their findings, the authors concluded that CFX DPI was well-tolerated and holds the ability to be an efficacious therapeutic alternative for individuals with bronchiectasis [118]. Apart from this, Timothy et al. [119] conducted a phase III trial assessing CFX DPI in bronchiectasis. Their research focused on examining the safety and effectiveness of this inhalation method among patients with bronchiectasis, a history of two or multiple worsening in the prior year, and specific sputum bacteria. Throughout the study, exacerbation rates remained low among all treatment groups. The active treatment displayed tendencies toward prolonging the duration before the initial exacerbation and reducing the frequency of exacerbations, although these outcomes did not reach statistical significance. Notably, CFX DPI was well-tolerated. While the study revealed trends toward clinical benefits associated with CFX DPI, the primary endpoints were not conclusively achieved [119]. Additional work by Bilton et al. [120] involved a double-blind, randomized, active comparator assessment to investigate the impact of adding an inhaled tobramycin solution to oral CFX for treating acute exacerbations in bronchiectasis patients with *P. aeruginosa* infection. Their research revealed that using inhaled CFX with tobramycin, when compared to a placebo, led to a superior antibacterial response. However, no statistically notable variance in clinical effectiveness was observed at the end of the 21-day cure assessment; however, both clinical and microbiological outcomes were consistent when inhaled tobramycin solution was included in conjunction with CFX compared to the placebo group [120]. Additionally, Bilton et al. [121] conducted another double-blind, placebo-controlled study (ORBIT 1), involving multiple centers to assess the effectiveness, safety, and tolerability of OD CFX for inhalation in treating *P. aeruginosa* infections in individuals with bronchiectasis. In a subsequent open-label study with 36 bronchiectasis patients, CFX DPI was well-tolerated. Both doses (150 mg and 100 mg CFX) exhibited significant mean reductions from baseline in *P. aeruginosa* colony forming units (CFUs) at 28 days, with decreases of 3.5 log10 (*p* < 0.001) and 4.0 log10 (*p* < 0.001), respectively, in the per protocol (PP) population [121]. The overall summary of CFX-loaded inhalable formulations against LRTIs in clinical settings is summarized in Table 2.

## 4. Challenges and Recent Advances

Inhalation treatment has been identified as the most efficient remedy for the specified respiratory bacterial infestations, allowing direct administration to the affected area. Consequently, inhaled antibiotics have been linked to decreased exacerbation frequency, significantly lowered bacterial levels in the airways, improved lung function recovery, and notably enhanced the well-being of individuals with pulmonary infections. However, existing inhaled antibiotics are not exhibiting optimal effectiveness in eliminating bacterial infections due to the persistent limitations and challenges they encounter [122]. Improving the lung-based absorption of inhaled CFX relies on its adequate solubility in water. This necessity is emphasized by the limited volume of pulmonary lining fluid (approximately 150 mL), distributed thinly (not exceeding 30 µm) across a substantial epithelial surface area (ranging from 140 to 160 m^2^) [123,124]. Concurrently, the relatively diminished effectiveness of current inhaled anti-infectives demands the administration of notably high doses, sometimes reaching several hundred milligrams. Hence, the water solubility of inhaled CFX is crucial, highlighting the urgency to develop strategies that significantly enhance its solubility in water [125]. Primarily, the poor bioavailability of CFX at the infected site, resulting in concentrations lower in the MIC, can expedite the emergence of resistance [122]. Recent clinical investigations have demonstrated that existing inhaled antibiotic compositions excel in halting the pathogen’s dissemination and limiting damage to the airway tissues but fall short of completely eliminating the infection [126]. The acidic conditions prevalent in the infected surroundings and biofilm can cause protonation of medications such as CFX, intensifying their interaction with alginate within the biofilm through charge-based reactions. This interaction subsequently diminishes the concentration of free drugs available at the intended site of action [122]. As a result, antibiotic levels might not surpass the MIC, fostering micro-environmental pressures that further encourage biofilm development and the emergence of drug-resistant bacterial subgroups [122]. Furthering this, the solid state of CFX exhibits a robust crystal lattice attributed to the interaction between the deprotonated carboxylate group and the protonated piperazine group, forming orderly molecular sequences over long distances. In contrast, the amorphous form of CFX comprises randomly distributed molecules with elevated free energy and a crystallization propensity [127]. This crystallization induces morphological transformations in the particles, consequently affecting their aerosol performance. Additionally, the amorphous state tends to absorb moisture in humid conditions, heightening inter-particulate capillary forces and compromising the aerosolization of particles [128]. As a result, the effectiveness of CFX against LRTIs diminishes, given that the effectiveness of the aerosolized drug relies on both the deposited dose at the target site and its distribution within the lungs [129]. Besides, utilizing inhaled CFX can decrease the required dosage and minimize systemic side effects, potentially benefiting pediatric treatment with this antibiotic. However, despite the antibiotic being concentrated in the lungs, it must still circumvent the mucus hurdle to reach its intended target. In the infected lungs of CF patients, bacteria colonize within the mucus, posing a substantial challenge in effectively treating pulmonary infections via inhaled medication [87]. Ultimately, there might be constraints associated with the utilization of inhaled CFX in conditions like COPD, bronchiectasis, or CF. These limitations could stem from challenges such as the impaired penetrability of CFX to reach smaller airways, the potential for the progression of drug-resistant strains, and the financial implications of therapy. It is imperative to acknowledge and tackle these concerns in the subsequent stages of clinical development [130].

Despite these constraints, significant advancements have been achieved in developing inhalable CFX dry powder over the past two decades. The narrative surrounding CFX provides valuable insights into the necessary approach for developing pharmaceutical formulations. Specifically, it emphasizes the importance of employing a rational formulation development strategy based on deep comprehension of the obstacles [131]. To the best of our understanding, there have been no prior reports on the combinational effectiveness of phage and dry powder antibiotics within the lung. Lin et al. [132] recently formulated an inhalable powder by simultaneously drying *Pseudomonas* phage PEV20 with CFX. Their study proposed to analyze the effect of this powder in vivo, utilizing a neutropenic mouse model of acute lung infection. Their findings indicated a substantial reduction in the bacterial burden of the clinical *P. aeruginosa* strain in the lungs of mice when treated with the combination PEV20-CFX powder. Conversely, no significant decline in bacterial density was noticed when the animals were treated solely with PEV20 or CFX. Examination of the lung’s immune responses revealed decreased inflammation associated with the bactericidal effectiveness of the PEV20-CFX powder. In summary, this finding showcases the combinatorial PEV20-CFX powder as a promising strategy for combating *P. aeruginosa* respiratory infections [132]. Bronchiectasis is a persistent lung condition characterized by thick, sticky mucus on the respiratory lining, hindering the effectiveness of inhaled medications. Ling et al. [133] recently developed inhalable microparticles containing CFX using silk fibroin and mannitol to address this challenge. Silk fibroin enhanced CFX’s capacity through robust electrostatic interactions, while mannitol facilitated drug penetration through the mucus, ensuring targeted delivery before clearance. Their study, encompassing both in vitro and in vivo experiments, exhibited that CFX microparticles did not impair lung activity or provoke the release of inflammatory cytokines in the lungs, showcasing exceptional biocompatibility and safety. As a result, CFX microparticles exhibit promise as a viable pulmonary drug delivery system for treating bronchiectasis [133]. Apart from this, several recent clinical studies have demonstrated that CFX DPI effectively lowered the occurrence of acute exacerbations in patients with LRTIs who were colonized with respiratory bacterial pathogens and lowered *P. aeruginosa* density in sputum. The safety profile of CFX DPI was found to be favorable. Additionally, following inhalation of CFX, no instances of bronchospasm or clinically notable alterations in lung function were observed, and systemic exposure to CFX remained low. This suggests a promising role for inhaled CFX in managing LRTIs [19,134,135]. Moreover, it is also reported that both formulations of CFX (CFX DPI and DRCFI) demonstrate bactericidal efficaciousness, not only against *P. aeruginosa* but also against other bacterial strains frequently found in individuals with LRTIs, including *M. catarrhalis* or *H. influenzae*. This remedial benefit could be harnessed for persistent infections linked to conditions like asthma or chronic bronchitis, provided their administration is carefully directed [23].

## 5. Safety, Tolerability, and Regulatory Concerns

Studies assessing the safety of CFX DPI have demonstrated its general tolerability. Multiple phase I and II trials in individuals with conditions like COPD, CF, or bronchiectasis suggest favorable tolerability, with phase III trials pending to confirm these findings [17,18,19]. In phase II trials, adverse events emerging during treatment were comparable between CFX DPI and placebo groups in terms of nature and frequency. Predominantly, patients using CFX DPI reported an unusual taste or dysgeusia, a property of the drug [23,116]. Notably, the low incidence of respiratory tract irritation observed in CFX DPI studies is significant given that coughing, bronchospasm, and airway blockages are potential concerns for existing and prospective inhalation interventions. In a recent assessment investigating the use of aztreonam for inhalation solution, serious adverse events like coughing and bronchospasm were observed in a small fraction of patients—one out of one hundred thirty-four for cough development and three out of one hundred thirty-four for bronchospasm [136]. Among the one hundred fifty-three patients who received treatment with CFX DPI across two studies (one involving bronchiectasis and the other CF patients), only six individuals reported bronchospasm [23,116]. In the CF study, the cough occurrence was 3.2% among those treated with CFX DPI compared to 10.8% in the placebo group [116]. Similarly, in the bronchiectasis study, the rates were 0% for CFX DPI and 7.8% for the placebo group [23]. These figures present a more favorable outcome compared to other DPI products like the tobramycin inhalation Podhaler, where a cough is considered a more prevalent issue [137].

In April 2014, the office of the orphan products development at the US Food and Drug Administration (FDA) provided orphan drug designation (ODD) to CFX DPI for addressing bronchiectasis. Furthermore, in November 2014, the FDA approved the Qualified Infectious Disease Product (QIDP) status for CFX DPI. QIDP designation is given to antimicrobial medications developed for treating severe and life-threatening infections, offering benefits such as accelerated approval, expedited evaluation by the FDA, and a prolonged period of market exclusiveness [14].

## 6. Future Perspectives

CFX DPI is being developed for prolonged, intermittent treatment to decrease exacerbations in patients of LRTIs with respiratory pathogens. It has been clinically observed that CFX DPI is well-tolerated with no significant detrimental impacts on lung function [13,20]. However, there is still concern about the tolerability of these inhaled CFX forms (CFX DPI and DRCFI), necessitating further exploration before their integration into clinical practice [23]. Animal studies and post-market monitoring will be crucial to assess the potential long-term toxicity risks associated with inhaled CFX [138]. Moreover, the existing data do not conclusively support the utilization of inhaled CFX in bronchiectasis patients experiencing acute pulmonary exacerbations. While preliminary clinical data on CFX DPI and DRCFI show efficacy among stable bronchiectasis patients with chronic respiratory pathogen colonization or infection, comprehensive information from fully published clinical trials is required to accurately determine the applicability and complete therapeutic benefits of inhaled CFX therapy [138]. Although advancements have been made in inhaled antibiotics for bronchiectasis over recent decades, there remain three significant unresolved matters necessitating additional exploration: determining the suitable patient cohort, establishing the optimal treatment duration, and accurately assessing the risk of antibiotic resistance. To address these inquiries and ascertain the most efficient treatment for individuals with this persistent condition, further multicenter randomized-controlled studies are essential [24].

In order to gain widespread acceptance, it will be essential to conduct cost-effectiveness analyses and enhance our comprehension of the development and consequences of CFX resistance over extended treatment periods. Existing research lacks the necessary statistical findings to demonstrate mortality advantages, making post-marketing observational studies vital for clinicians. Additionally, it is crucial to undertake studies to ascertain the long-term ‘eradication’ of *Pseudomonas* [14]. Moreover, physicians would like to witness broader inclusion of bronchiectasis phenotypes in CFX DPI studies, as current RESPIRE (largest placebo-controlled, double-blind phase III trial program in bronchiectasis) studies encompass only about 40–60% of likely causative agents of bronchiectasis. A considerable number of patients also exhibit moderate to severe COPD, and the overlap between bronchiectasis and COPD is linked to a worse prognosis compared to idiopathic bronchiectasis. Consequently, investigations into the efficacy of CFX DPI in BCOS are anticipated with great interest [14]. Furthering this, persistent airway infections caused by *P. aeruginosa* are recognized in CF and can manifest at any age, regardless of the extent of lung activity degradation. In contrast, chronic infections exist in other conditions like COPD or bronchiectasis but lack well-defined characteristics and standardized treatments. Therefore, in the clinical application of CFX, it is crucial to identify and address persistent infections to diminish the bacterial reservoir capable of triggering subsequent exacerbations in patients of all ages. This approach also aims to minimize local inflammation, which could hasten the reduction in lung function [130].

Developing an inhaled liposomal formulation presents greater complexities compared to conventional inhaled nebulizer solutions. Numerous obstacles must be tackled, including ensuring the consistency of fabricating for each liposome batch, maintaining formulation stability throughout its shelf-life, enduring stability during the aerosolization process, achieving an optimal aerosol particle size distribution for effective airway deposition, and facilitating drug release from the liposomes at a suitable rate to sustain drug proportion higher the MIC until the subsequent delivery [139].

## 7. Expert Opinion

Achieving optimal PK through pulmonary drug delivery is a complex process impacted by various factors which include the type of delivery device used, the drug’s physicochemical properties, particle-related aspects, and patient-specific factors. Research indicates that the deposition of inhaled antibiotics in the central airways is greater when bronchial obstruction and mucus plugging are increased. Conversely, the peripheral, diseased regions of the lungs receive lower antibiotic doses compared to healthier areas. As a result, patients with more advanced lung disease may require higher drug doses to attain adequate concentrations throughout the lung, particularly in affected areas [140,141]. Prolonged use of antibiotics raises the concern of CFX-resistant pathogens emerging. Evaluating this risk in clinical settings requires follow-up periods exceeding 8 weeks. Additionally, an important consideration is the lack of evidence supporting enhancement in the well-being of patients in studies, despite being a secondary endpoint. Despite advancements in inhaled antibiotics for LRTIs over recent decades, three key issues remain unresolved and warrant further investigation: identifying the appropriate patient population, determining the optimal treatment duration, and assessing the true risk of antibiotic resistance. More multicenter randomized controlled trials are necessary to address these concerns and establish the most appropriate therapy for this chronic condition [24]. Finally, we recommend carrying out in-depth investigations into the efficacy of CFX DPI in BCOS prior to inhibiting the poor prognosis. Current data on the effectiveness of inhaled CFX for LRTIs are promising. However, more research is required to identify the optimal target population and the ideal treatment duration.

## 8. Conclusions

The emergence of inhaled CFX shows promise for effectively managing chronic LRTIs. As we elaborated in this review, inhaled CFX treatment demonstrated superior efficacy than free drug in several phases of clinical trials and in preclinical settings. Further exploration is needed to address three key unresolved issues: identifying the right patient group, determining the optimal treatment duration, and accurately assessing the risk of antibiotic resistance. Moreover, inhalable powder of *Pseudomonas* phage PEV20 with CFX revealed a substantial reduction in the bacterial burden of the clinical *P. aeruginosa* strain in the lungs of mice and reduced inflammation. Recently performed research indicates that an increased biosynthesis of fatty acids plays a crucial part in the progression of CFX resistance in *P. aeruginosa*. Importantly, a crucial focus lies in investigations into the effectiveness of CFX DPI in bronchiectasis and COPD, aiming to reduce prognostic overlap between the two conditions. Within the infected lungs of CF/COPD patients, bacteria are residing within the mucus/biofilms, and the effective management of pulmonary infections could be achieved through inhaled medications. Therefore, forthcoming research on inhaled CFX should strongly emphasize strategies to circumvent the hindrance of mucus and reach its intended target.

## Figures and Tables

**Table 1:** Summary of CFX-loaded inhalable formulations against LRTIs in preclinical settings.

Drug Incorporated	Formulation Type	Bacterial Strain	Major Findings	Ref.
CFX and colistin	Liposomal powder	*P. aeruginosa*	Remarkable antibacterial effectiveness	[97]
CFX and colistin	Liposomal powder	*P. aeruginosa*	Enhanced drug retention on the lung epithelial surfaces	[98]
CFX	Nanostructured lipid carriers		Significant amelioration in the FPF and higher FPD	[99]
CFX	Silver nanoparticles	*P. aeruginosa*	Highly efficacious against impeding growth and biofilm formation	[100]
CFX	Dry powder	*P. aeruginosa*	Markedly higher antibacterial effect	[101]
CFX	Dry powder		Considerably higher mucus permeation and greater antibacterial effectiveness	[102]
CFX	PLGA nanoparticles	*P. aeruginosa*	Improved effectiveness and noticeable reduction in mucus turbidity	[103]
CFX	Liposome	*Yersinia pestis* (Murine model of Pneumonic plaque)	Notable enhancement in survival Reduced bacterial load in the lungs and spleens	[104]
CFX	Liposome		Effectively regulated drug release	[58]
CFX	Dry powder		Demonstrated superior performance in terms of aerosolization and increased drug content	[50]
CFX-Cu^2+^	Microparticles	*P. aeruginosa* (Rat model)	Significant reduction of lung burden of bacteria	[105]
CFX	Microparticles	*P. aeruginosa* *S. aureus*	Sustained drug levels in the lungs to obtain therapeutic effects	[106]
CFX	Spray-dried powder	Rat model	Exhibited rapid drug release rate and higher in vivo lung exposure	[6]
CFX	Nanoparticles		FPF enhanced proportionally with higher drug loading Proposed PEtOx is a potential carrier for LRTIs	[1]
CFX and phage	Dry powder	*P. aeruginosa*	Demonstrated excellent synergistic antimicrobial killing efficacy and preserved bactericidal synergy	[107]
CFX	Sub-micron particles	*P. aeruginosa* and *S. aureus*	Revealed effective antimicrobial action Particles were biocompatible with A549	[108]
CFX	Microparticle-based dry powder		Ameliorated aerosol performance	[109]
CFX and PMB	Dry powder	*P. aeruginosa*	Maintained their ability to combat bacterial strain Good stability in solid-state form and suitable aerodynamic properties	[110]
CFX and PMB	Dry powder	*A. baumannii*	Efficaciously inhibited the emergence of resistance	[111]
CFX and curcumin	Nanosuspension-based dry powder	*P. aeruginosa* and *S. aureus*	Effective antibacterial effects and excellent release properties	[72]
CFX	Liposome	*Rat model* (Lipopolysaccharide-induced pneumonia)	CFX concentration in both AMs and lung ELF followed significantly higher time-courses compared to free CFX CFX concentration in plasma is lower than AMs and ELF	[112]
CFX and colistin	Dry powder		Elevated physical stability and aerosolization of amorphous inhalable CFX	[113]
CFX with acetylcysteine and dornase alfa	Dry powder		Improved dissolution rates than untreated CFX	[114]
CFX and quercetin	Spray-dried particles		Improved stability and better aerosol performance	[115]

**Table 2:** CFX-encapsulated inhalable formulations against LRTIs in clinical settings.

Inhalable Formulation Type	Study Type	Disease Category	Major Findings	Ref.
Dry powder	Phase II, randomized double-blind, multicenter	Bronchiectasis	Well tolerated and remarkable reduction in total bacterial load	[23]
Dry powder	Phase I, single-dose, randomized, placebo-controlled	Chronic pulmonary infections	Well-tolerated with no detrimental implications on lung function evidenced clinically	[20]
Dry powder	Phase I, randomized, single-dose, dose-escalation	Cystic fibrosis	Well-tolerated and no instances of mortality	[19]
Dry powder	Phase I, randomized, dose-escalation	Cystic fibrosis	No serious detrimental effects, no notable changes in lung function measurements, and quickly absorbed	[18]
Dry powder	Phase I, randomized, single-blind design	Moderate to severe COPD	No occurrences of serious or severe detrimental effects, well-tolerated Elevated drug levels achieved in sputum	[17]
Liposome	Two phase III, randomized	Bronchiectasis and chronic lung infection	Significantly higher median duration than placebo group	[94]
Liposome	Phase II, randomized, double-blind, and placebo-controlled	Bronchiectasis	Decline in *P. aeruginosa* burden by day 28 and better tolerated	[91]
Dry powder	Placebo-controlled, randomized, phase IIb	Cystic fibrosis	No remarkable variations in primary efficacy endpoint but significant reduction in FEV1 reduction than placebo Positive impacts on sputum bacterial burden and well-being	[116]
Liposome	Two phase III, randomized	Bronchiectasis	Significant improvements in respiratory symptoms	[117]
Dry powder	Phase III, double-blind, placebo-controlled	Bronchiectasis	Remarkably extended the duration until the initial exacerbation than placebo Diminished the incidence of exacerbations	[118]
Dry powder	Phase III, placebo-controlled, randomized trial	Bronchiectasis	Revealed tendencies toward extending the duration before the initial exacerbation, diminished repetition of exacerbations, and well-tolerated	[119]
Solution (Tobramycin + CFX)	Double-blind, randomized, active comparator, parallel design	Bronchiectasis	Demonstrated greater microbiological response (against *P. aeruginosa*)	[120]
Dry powder	Multicenter, randomized, double-blind, placebo-controlled	Bronchiectasis	Well-tolerated and considerable mean reductions from baseline in *P. aeruginosa* CFUs at 28 days	[121]
